# Case for diagnosis. Infraorbital schwannoma^☆^^☆☆^

**DOI:** 10.1016/j.abd.2020.08.014

**Published:** 2021-03-16

**Authors:** Irving Llibrán Reyna-Rodríguez, Sonia Chavez-Alvarez, Jorge Ocampo-Candiani

**Affiliations:** Dermatology Department, Faculty of Medicine, Hospital Universitario “Dr. Jose E. Gonzalez”, Universidad Autonoma de Nuevo Leon, Monterrey, Mexico

**Keywords:** Infraorbital schwannoma, Neurilemmoma, Schwannoma, Tumor

## Abstract

We report a 40-year-old man, with an unremarkable personal and family history, who presented for evaluation of an asymptomatic papule located on his right cheek. Histopathology revealed an encapsulated neoplasm within the dermis; composed by narrow, elongated, and wavy cells with an ill-defined cytoplasm, dense chromatin and tapered ends interspersed with collagen fibers. Pathologic findings were consistent with tissue of Antoni B pattern. The diagnosis was an infraorbital schwannoma. The incidental finding of rare tumors like this, should make clinicians consider a greater spectrum of differential diagnoses for a unilateral skin-colored papule on the cheek of patients.

## Case report

**Figure 1:**
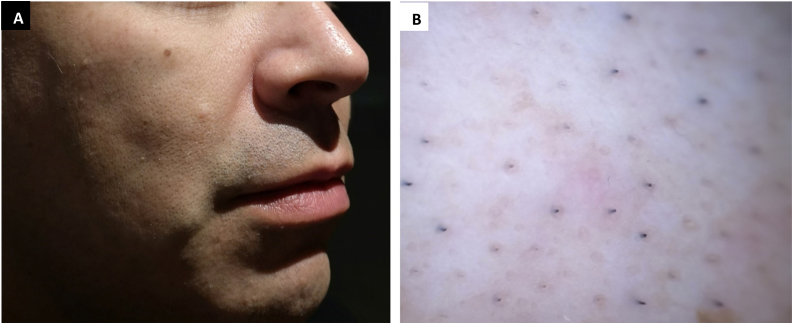
(A) Skin-colored papule on the right cheek. (B) Polarized dermoscopic image showing non-characteristic features.

A 40-year-old male, with an unremarkable personal and family history, presented for evaluation of an asymptomatic papule located on his right cheek that had been present since childhood. He had not received any treatment. Physical examination revealed a solitary, circumscribed, firm, skin-colored, 4–6 mm papule (Fig. 1A). Dermoscopic (polarized mode, Dermlite) examination demonstrated non-specific erythema, normal pigmentary network without follicular plugging (Fig. 1B). An excisional biopsy of the lesion was performed.

**Figure 2:**
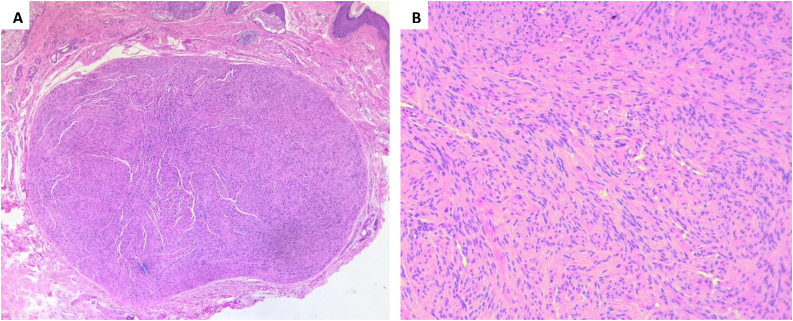
(A) A well-defined encapsulated tumor in the dermis (Hematoxylin & eosin, ×100). (B) Spindle Schwann cells with Verocay bodies, Anthony B pattern (Hematoxylin & eosin, ×200).

**Figure 3:**
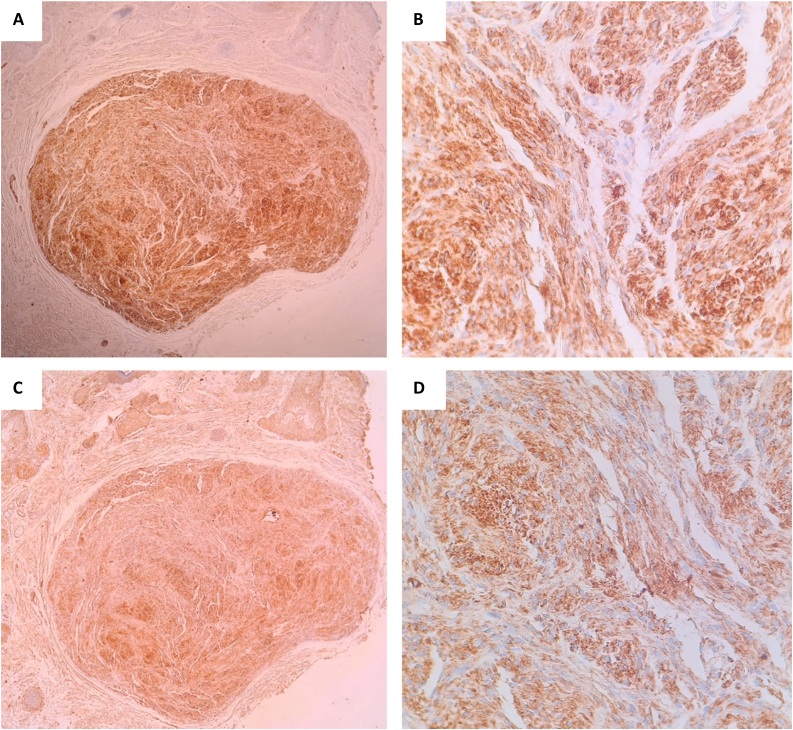
(A and B) Positive immunohistochemistry exam for S100. (C and D) Positive immunohistochemistry exam for GFAP.

Histopathology (Fig. 2 A and B) revealed an encapsulated neoplasm within the dermis; on higher magnification, we could see narrow, elongated, and wavy cells with an ill-defined cytoplasm, dense chromatin and tapered ends interspersed with collagen fibers. Immunohistochemical stains for S-100 and (Fig. 3 A and B) and Glial Fibrillary Acidic Protein (GFAP) (Fig. 3 C and D) were positive. Pathologic findings were consistent with tissue of Antoni B pattern. The patient had good healing of the site of excisional biopsy.

## What is your diagnosis?

a) Dermal nevus
b) Sebaceous hyperplasia
c) Infraorbital schwannoma
d) Trichoepithelioma

## Discussion

Schwannomas, also called neurilemmomas, are benign ectodermal neoplasms originated from peripheral, cranial, or autonomic nerve Schwann cell sheaths.^1^ Male: Female ratio is 1:1 but some authors consider that women are more affected.^2^, ^3^ Most of the extracranial schwannomas are present in the head and neck (25%–45%).^4^, ^5^ These can develop from any of the 12 cranial nerves (mainly auditory nerve); except optic and olfactory (because they lack Schwann cells in their sheaths).^4^ The trigeminal nerve is rarely associated with schwannomas; especially in the infraorbital nerve area. ^3^ They present as a slow-growing mass, without pain or a neurological deficit.^4^ Diagnosis is made with histopathology and clinical correlation. Histopathologically, Schwann cells can be seen as spindle-shaped cells in parallel rows forming a typical palisading pattern of Verocay bodies. Classification is based on cellularity: Antoni A (hypocellular) or Antoni B (hypercellular).^6^ No fibroblasts, mast cells, neurilemmal, or endoneurial cells are present.^3^ Possible clinical differential diagnoses in this location include dermal nevus, dermoid cyst, sebaceous hyperplasia, trichoepithelioma, and palisaded encapsulated neuroma (PEN). On histopathology the principal differential diagnosis is PEN (contains axons), both tumors are S-100 positive, schwannoma is positive for GFAP and negative for neurofilament while PEN is just the opposite.^7^ Other less possible histopathological differential diagnoses are neurofibroma (lacks a capsule, contains mucopolysaccharide ground substance and fewer axons with myelin sheaths), traumatic neuroma (axonal and Schwann cells in addition to scarring and inflammatory cells), and leiomyoma (spindle cell lesion composed of muscle cells).^7^, ^8^ Extracranial schwannomas have a good prognosis with exclusively surgical treatment (approach according to size, extent, and anatomical location).^9^ Malignant transformation is extremely rare when presenting as an isolated lesion.^10^ The incidental finding of rare tumors like this infraorbital nerve schwannoma should make clinicians consider a greater spectrum of differential diagnosis for a unilateral skin-colored papule on the cheek of healthy middle-age patients.

## Financial support

None declared.

## Authors' contributions

Irving Llibrán Reyna-Rodríguez: Study conception and planning; preparation and writing of the manuscript; data collection, analysis, and interpretation; critical literature review.

Sonia Chavez-Alvarez: Study conception and planning; critical literature review; effective participation in research orientation; approval of the final version of the manuscript.

Jorge Ocampo-Candiani: Study conception and planning; critical literature review; effective participation in research orientation; approval of the final version of the manuscript.

## Conflicts of interest

None declared.
